# Synthesis and *In-vitro* Antibacterial Activities of Acetylanthracene and Acetylphenanthrene Derivatives of Some Fluoroquinolones

**Published:** 2011

**Authors:** Fazel Shamsa, Alireza Foroumadi, Hashim Shamsa, Nasrin Samadi, Mohammad Ali Faramarzi, Abbas Shafiee

**Affiliations:** a *Department of Medicinal Chemistry, Faculty of Pharmacy and Pharmaceutical Sciences Research Center, Tehran University of Medical Sciences, Tehran, Iran.*; b *Drug Design and Development Research Center, Tehran University of Medical Sciences, Tehran, Iran.*; c *Department of Drug and Food Control, Faculty of Pharmacy, Tehran University of Medical Sciences, Tehran, Iran.*; d *Department of Pharmaceutical Biotechnology, Faculty of Pharmacy, Tehran University of Medical Sciences, Tehran, Iran.*

**Keywords:** *N*-substituted piperazinyl quinolones, Anthracene derivatives, Phenanthrene derivatives, *In-vitro *antibacterial activity

## Abstract

Novel analogues of *N*-piperazinyl fluoroquinolones were prepared and evaluated against a panel of Gram-positive and Gram-negative bacteria, to study the effect of introducing bulky anthracene and phenanthrene moieties on the antibacterial effects of norfloxacin, ciprofloxacin and gatifloxacin. Although most of the novel synthesized compounds had lower antibacterial effects, some derivatives showed better activity in comparison with mother drugs based on molar concentration; for example, the 3-acetyl phenanthrene analogue of norfloxacin was more effective than *E. coli *and *K. pneumonia*.

## Introduction

**Figure 1:**
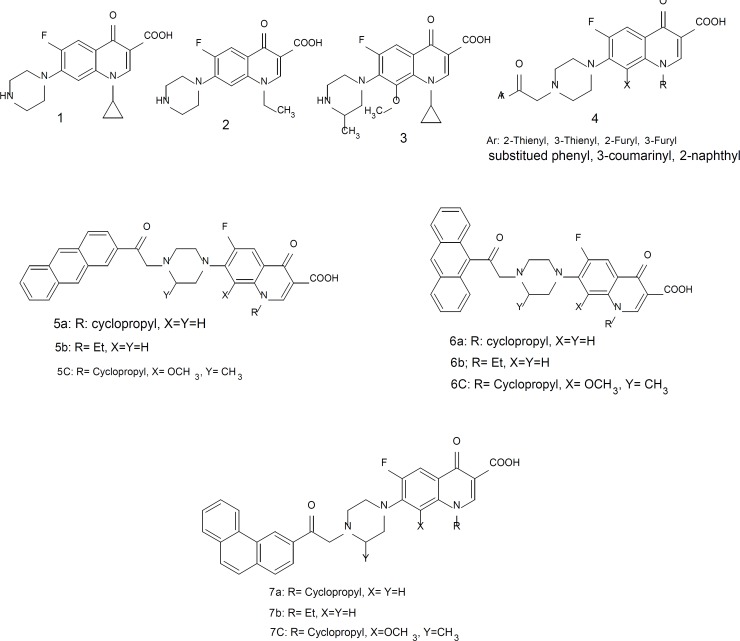
The change of the content of the monoterpenes and sesquiterpenes from pre-flowering to post-flowering stage

Quinolones are a group of synthetic antibacterial agents structurally related to nalidixic acid (1). Nalidixic acid was the first introduced quinolone for the treatment of urinary tract infections caused by Gram-negative organisms (2, 3). Since the discovery of nalidixic acid, more than 10000 analogues have been synthesized and their antibacterial activities were evaluated (4). Fluorination of the quinolones at C-6 position and introduction of piperazine ring at C-7 position by Koga and colleagues (5) led to the evolution of fluoroquinolones, *i.e*. ciprofloxacin 1, norfloxacin 2, and gatifloxacin 3 (Figure 1), as new broad spectrum antibacterial drugs with better antibacterial and pharmacokinetic profiles (6). The main mechanism of these drugs is in the inhibition of DNA gyrase and topoisomerase IV (7). It was shown that DNA gyrase inhibition and the entrance into the microbial cells significantly depend on the groups of C-7 (8, 9). Some fluoroquinolone derivatives with thiophene (10, 11), furan (12), substituted phenyl (13) and coumarin (14) attached to the piperazine ring at 7-position were synthesized with better antibacterial effects against Gram-positive bacteria (Figure 1.1-1.4). Recently, the synthesis of some *N*-[2-(2-naphthyl)ethyl] piperazinyl quinolones (Figure 1.4, Ar = 2-naphthyl) with potent antibacterial activity against Gram-positive and Gram-negative bacteria, have been reported (15). In the present study, some novel analogues of fluoroquinolones such as ciprofloxacin 1, norfloxacin 2, and gatifloxacin 3, with 2-oxo-2-(anthracene-2-yl) ethyl (5a-c), 2-oxo-2-(anthracene-9-yl) ethyl (6a-c) and 2-oxo-2-(phenanthrene-2-yl) ethyl (7a-c) have been synthesized to study the effect of initiating bulky anthracene and phenanthrene moieties on the antibacterial activities against Gram-positive and Gram-negative bacteria.

## Experimental

*Chemistry*

All solvents and the chemicals used in this study were purchased from Merck Co. (Merck, Germany) and Aldrich chemicals agents. Melting points were determined on Electrothermal 9100 apparatus and are uncorrected. The IR spectra were obtained on a Shimadzu 470 spectrophotometer (KBr disks). ^1^H-NMR spectra were measured using 80 MHz spectrometer and the chemical shifts are expressed as δ (ppm) with tetramethylsilane as internal standard. Elemental analyses were within ± 0.4% of theoretical values for C, H and N. 

*General procedure for the peparation of 7-[4-(2-aryl-2-oxoethyl)-1-piperazinyl] quinolones 5-7*

A mixture of 2-bromo-1-(anthracen-2-yl) ethanone (9a), 2-bromo-1-(anthracen-9-yl) ethanone (9b) or 2-bromo-1-(phenanthren-2-yl) ethanone (9c) (0.33 mmol), fluoroquinolone (1, 2 or 3) (0.3 mM) and NaHCO_3_ (0.3 mM) in DMF (5 mL), was stirred at 45°C for 48-72 h. After the consumption of fluoroquinolone, water (30 mL) was added and the precipitate was filtered, washed with water and crystallized from EtOH-CHCl_3_ to give the target compounds (Figure 1.5-1.7). 

*1-Cyclopropyl-6-fluoro-1, 4-dihydro-7-[4-[2-(anthracen-2-yl)-2-oxoethyl] piperazin-1-yl]-4-oxo-3-quinoline carboxylic acid (5a)*

Yield: 60%; m.p. 163-164°C; IR (KBr, cm^-1 ^) υ max: 1622, 1680 and 1728 (C = O); ^1^H-NMR (DMSO-d6) δ : 1.12-1.33 (m, 4 H, cyclopropyl), 2.81-3.14 (m, 4 H, piperazine), 3.30-3.70 (m, 4 H, piperazine), 3.72-3.86 (m, 1 H, cyclopropyl), 4.22 (s, 2 H, COCH_2_), 7.29-7.60 (m, 5 H, aromatic), 7.90-8.25 (m, 4 H, aromatic), 8.45-8.70 (m, 2 H, aromatic), 8.76 (s, 1 H, H-2 quinolone). MS: m/z (rel. Int. %): 549.1 (M^+^, 4), 504.3 (10), 330.4 (12), 285.2 (8), 221.2 (20), 178 (100). Anal. (C_33_H_28_FN_3_O_4_) C, H and N.

*1-Cyclopropyl-6-fluoro-1, 4-dihydro-7-[4-[2-(anthracen-9-yl)-2-oxoethyl] piperazin-1-yl]-4-oxo-3-quinoline carboxylic acid (5b)*

Yield: 75%; m.p. 221-223ºC; IR (KBr, cm^-1^ ) υ max: 1624, 1681 and 1730 (C = O); ^1^H-NMR (DMSO-d6) δ : 1.19-1.38 (m, 4 H, cyclopropyl), 2.81-2.93 (m, 4 H, piperazine), 3.25-3.41 (m, 4 H, piperazine), 3.60-3.90 (m, 3 H, 1 H cyclopropyl and 2 H COCH_2_), 7.29-7.65 (m, 5 H, aromatic), 7.96-8.20 (m, 4 H, aromatic), 8.52-8.68 (m, 2 H, aromatic), 8.72 (s, 1 H, H-2 quinolone). MS: m/z (rel. Int. %): 549 (M^+^, 6), 504 (12), 330 (11), 285 (10), 221 (21), 178 (100). Anal. ( C_33_H_28_FN_3_O_4_) C, H and N.

*1-Cyclopropyl-6-fluoro-1, 4-dihydro-7-[4-[2-(phenanthren-2-yl)-2-oxoethyl]piperazin-1-yl]-4-oxo-3-quinoline carboxylic acid (5c)*

Yield: 40%; m.p. 278-280ºC; IR (KBr, cm^-1^) υ max: 1630, 1682 and 1720 (C = O); ^1^H-NMR (DMSO-d6) δ : 1.10-1.39 (m, 4 H, cyclopropyl), 2.81-3.10 (m, 4 H, piperazine), 3.32-3.64 (m, 4 H, piperazine), 3.72-3.84 (m, 1 H, cyclopropyl), 4.12 (s, 2 H, COCH2), 7.40-7.85 (m, 5 H, aromatic), 7.90-8.25 (m, 4 H, aromatic), 8.50-8.65 (m, 2 H, aromatic), 8.75 (s, 1 H, H-2 quinolone). MS: m/z (rel. Int. %): 549 (M+, 4), 504 (11), 330 (12), 285 (10), 221 (22), 178 (100). Anal. (C_33_H_28_FN_3_O_4_) C, H and N.

*1-Ethyl-6-fluoro-1, 4-dihydro-7-[4-[2-(anthracen-2-yl)-2-oxoethyl] piperazin-1-yl]-4-oxo-3-quinoline carboxylic acid (6a)*

Yield: 65%; m.p.: 217-219 °C; IR (KBr, cm -1 ) õ max: 1624, 1685 and 1725 (C = O), 3420 (OH); ^1^H-NMR (DMSO-d6) ä : 1.58 (t, 3 H, CH_3_, *J *= 7 Hz), 2.55-2.71 (m, 4 H, piperazine), 3.34-3.60 (m, 4 H, piperazine), 4.12 (s, 2 H, COCH2), 4.42 (q, 2 H, CH_2_-CH_3_, *J *= 7Hz), 6.95 (d, 1 H, H-8 quinolone, *J *= 7 Hz), 7.43-7.65 (m, 5 H, aromatic), 7.97-8.30 (m, 4H, aromatic), 8.41-8.60 (m, 1 H, aromatic), 8.76 (s, 1 H, H-2 quinolone). MS: m/z (rel. Int. %): 537 (M^+^, 6), 492 (8), 317 (13), 272 (9), 221 (21), 178 (100). Anal. (C_32_H_28_FN_3_O_4_) C, H and N.

*1-Ethyl-6-fluoro-1, 4-dihydro-7-[4-[2-(anthracen-9-yl)-2-oxoethyl] piperazin-1-yl]-4-oxo-3-quinoline carboxylic acid (6b)*

Yield: 50%; m.p.: 173-175ºC; IR (KBr, cm -1 ) õ max: 1621, 1685 and 1724 (C = O), 3420 (OH); ^1^H-NMR (DMSO-d6) ä : 1.60 (t, 3 H, CH3), 2.90-3.20 (m, 4 H, piperazine), 3.40-3.65 (m, 4 H, piperazine), 4.02 (s, 2 H, COCH2), 4.35 (q, 2 H, CH_2_-CH_3_), 6.90 (d, 1 H, H-8 quinolone, *J *= 7Hz), 7.35-7.65 (m, 5 H, aromatic), 7.70-8.20 (m, 4 H, aromatic), 8.01-8.15 (m, 1 H, aromatic), 8.72 (s, 1 H, H-2 quinolone). MS: m/z (rel. Int. %): 537 (M^+^, 7), 492 (10), 317 (8), 272 (10), 221 (24), 178 (100). Anal. (C_32_H_28_FN_3_O_4_) C, H and N.

*1-Ethyl-6-fluoro-1,4-dihydro-7-[4-[2-(phenanthren-2-yl)-2-oxoethyl]piperazin-1-yl]-4-oxo-3-quinoline carboxylic acid (6c)*

Yield: 50%; m.p.: 173-175°C; IR (KBr, cm -1 ) õ max: 1621, 1685 and 1724 (C = O), 3420 (OH); ^1^ H NMR (DMSO-d6) ä : 1.58 (t, 3 H, CH3), 2.85-3.10 (m, 4 H, piperazine), 3.35-3.61 (m, 4 H, piperazine), 4.15 (s, 2 H, COCH2), 4.32 (q, 2 H, CH_2_-CH_3_), 6.85 (d, 1 H, H-8 quinolone, *J *= 7 Hz), 7.40-7.85 (m, 4 H, aromatic), 7.90-8.25 (m, 4 H, aromatic), 8.50-8.65 (m, 2 H, aromatic), 8.75 (s, 1 H, H-2 quinolone). MS: m/z (rel. Int. %): 537 (M^+^, 7), 492 (10), 317 (8), 272 (10), 221 (24), 178 (100). Anal. (C_32_H_28_FN_3_O_4_) C, H and N. 

*1-Cyclopropyl-6-fluoro-7-[4-[2-(anthracen-2-yl)-2-oxoethyl] piperazin-1-yl]-8-methoxy-4-oxo-3-quinoline carboxylic acid (7a )*

Yield: 54%; m.p. 183-184°C; IR (KBr, cm^-1^ ) υ max: 1623, 1682 and 1732 (C = O); ^1^H-NMR (DMSO-d6) δ : 1.02 –1.60 (m, 7H, 4H cyclopropyl and 3H CH_3_-piperazine), 3.30-3.70 (m, 8 H, 7 H piperazine and 1 H cyclopropyl), 3.74 (s, 3 H, CH_3_O), 4.12 (s, 2 H, COCH_2_), 7.30-7.60 (m, 5 H, aromatic), 7.88-8.22 (m, 4 H, aromatic), 8.42-8.71 (m, 1 H, aromatic), 8.83 (s, 1 H, H-2 quinolone). MS: m/z (rel. Int. %): 593 (M^+^, 3), 548 (6), 372 (16), 327 (10), 221 (22), 178 (100). Anal. (C_35_H_32_FN_3_O_5_) C, H and N.

*1-Cyclopropyl-6-fluoro-7-[4-[2-(anthracen-9-yl)-2-oxoethyl] piperazin-1-yl]--8-methoxy-4-oxo-3-quinoline carboxylic acid (7b )*

Yield: 45%; m.p. 191-192°C; IR (KBr, cm^-1^ ) υ max: 1624, 1680 and 1725 (C = O); ^1^HNMR (DMSO-d6) δ : 0.95 –1.40 (m, 7 H, 4 H cyclopropyl and 3 H CH_3_-piperazine), 2.90-3.30 (m, 4 H, piperazine), 3.30-3.65 (m, 4 H, 3H piperazine and 1 H cyclopropyl), 3.79 (s, 3 H, CH_3_O), 4.02 (s, 2 H, COCH_2_), 7.52-7.55 (m, 5 H, aromatic), 8.00-8.30 (m, 4 H, aromatic), 8.50-8.63 (m, 1 H, aromatic), 8.76 (s, 1 H, H-2 quinolone). MS: m/z (rel. Int. %): 593 (M^+^, 5), 548 (10), 371 (11), 327 (8), 221 (20), 178 (100). Anal. (C_35_H_32_FN_3_O_5_) C, H and N.

*1-Cyclopropyl-6-fluoro-7-[4-[2- (phenanthren-2-yl)-2-oxoethyl] piperazin-1-yl]--8-methoxy-4-oxo-3-quinoline carboxylic acid (7c )*

Yield: 43%; m.p. 203-204 °C; IR (KBr, cm^-1 ^) υ max: 1620, 1681 and 1720 (C = O); ^1^H-NMR (DMSO-d6) δ : 1.00 –1.65 (m, 7 H, 4 H cyclopropyl and 3 H CH_3_-piperazine), 3.33-3.73 (m, 8 H, 7 H piperazine and 1 H cyclopropyl), 3.75 (s, 3 H, CH_3_O), 4.11 (s, 2 H, COCH2), 7.40-7.85 (m, 4 H, aromatic), 7.90-8.25 (m, 4 H, aromatic), 8.50-8.65 (m, 2 H, aromatic), 8.75 (s, 1 H, H-2 quinolone). MS: m/z (rel. Int. %): 593 (M^+^, 5), 548 (6), 371 (10), 327 (13), 221 (30), 178 (100). Anal. (C_35_H_32_FN_3_O_5_) C, H and N.

*Determination of the minimum inhibitory concentration (MIC)*

The MIC of the synthesized compounds (1a-3c) were determined by conventional agar dilution method (16, 17) with respect to different microorganism test including G-positive (S. *aureus *ATCC 6538p, *S. epidermidis *ATCC 12228, and Bacillus subtilis ATCC 6633) and G-negative (E. coli ATCC 8739, *K. pneumonia *ATCC 10031 and *P. aeruginosa *ATCC 9027) bacteria. Antimicrobial activities of compounds (1a-3c) were also examined against two clinical isolate methicillin-resistant *S. aureus *(MRSA I and II) in addition to the mentioned microorganism test. Two-fold dilution of the test compounds and the standard antibacterial agents, 1, 2, and 3 (Figure 1) were prepared in dimethyl sulfoxide (DMSO; 1 mL). Each dilute was added to molten Mueller-Hinton (MH) agar (19 mL) at 50°C to give a final concentration of 100, 50, 25, 12.5, 6.25, 3.125, 1.56, 0.78, 0.39, 0.195, 0.098, 0.049, 0.025, 0.012, 0.006 and 0.003 μg mL^-1^. The bacterial inocula were prepared by suspending overnight colonies from MH agar media in 0.85% saline. The inocula were adjusted photometrically at 600 nm to a cell density equivalent to approximately 0.5 McFarland standards (1.5 x 10^8^ CFU/mL). The suspensions were then diluted in 0.85% saline to make 10^7^ CFU/mL. The plates were spot-inoculated with 1 μL of bacterial suspensions (10^4^ CFU/spot); including a control plate containing 1 mL DMSO without any antibacterial agent. The plates were incubated at 35-37°C and examined after 18 h. The MIC was determined as the lowest concentration of the agent that completely inhibits visible growth of the microorganisms.

## Results and Discussion

**Figure 2:**
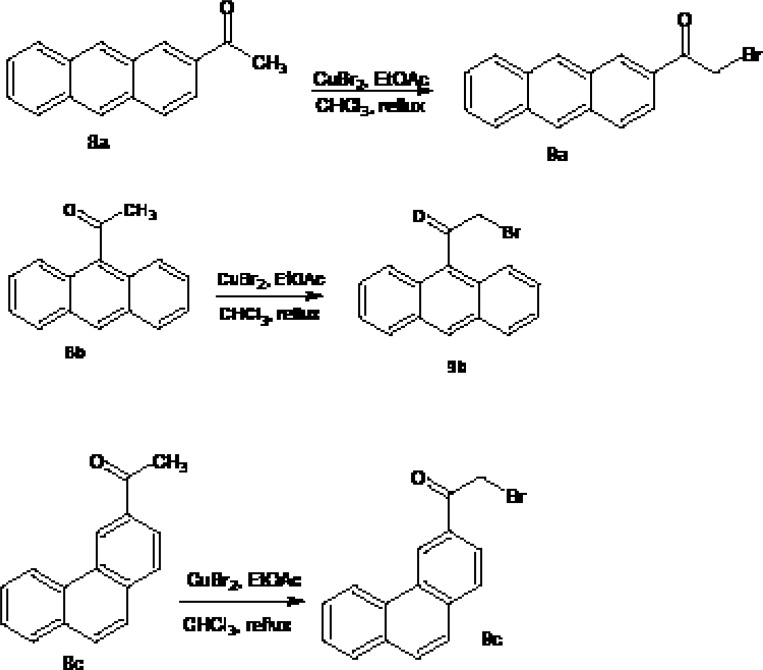
Synthesis of the intermediate compounds 9a-c

**Figure 3:**
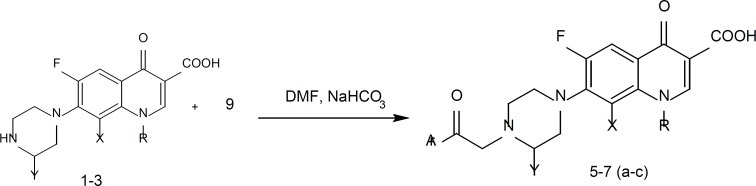
Synthesis of target compounds 5-7

The synthetic pathways to the intermediates (9a-c) and the target compounds (5-7) are presented in Figure 2 and 3. The acetyl derivatives of anthracene and phenanthrene (8a-c) were brominated with CuBr_2_ in refluxing CHCl_3_-EtOAc to give the corresponding α-bromoacetyl derivatives (9a-c). Reactions of fluoroquinolones 1-3 with compounds 9a, 9b or 9c in DMF in the presence of NaHCO_3_ at 45^°^C afforded corresponding 7-[4-(2-aryl-2-oxoethyl)-1-piperazinyl] quinolones 5-7. Compounds 5-7 (a-c) were tested against some gram-positive (*Staphylococcus aureu*s ATCC 6538P, *Staphylococcus epidermedis *ATCC 12228, *Bacillus subtlis *ATCC 66339) and Gram-negative bacteria (*Escherichia coli *ATCC 8739, *K. pneumonia*e 10031 and *Pseudomonas aeruginosa *ATCC 9027) using conventional agar-dilution method. 

The MIC (minimum inhibitory concentration) values were determined in comparison with the corresponding mother drugs (Table 1.). Through this table, it is concluded that the most sensitive bacteria is *K. pneumonia *which is inhibited by 1 (0.003 μg μg/mL), 2 (0.024 μg/mL) or 3 (0.006 μg/mL) and all synthesized compounds showed a good activity against this bacteria (MIC = 0.006-0.781μg mL^-1^). All target compounds 5-7, except compound for 5a, did not show good activity against *P. aeruginosa*. In most cases, the new synthesized compounds showed higher MIC values than the corresponding mother drugs against Gram-positive and Gram-negative bacteria; however, different activity profiles were observed among target compounds 5-7. Although most of the novel synthesized compounds had lower antibacterial effects, some derivatives showed better activity in comparison with the mother drugs based on molar concentration; for example, the 3-acetyl phenanthrene analogue of norfloxacin (6b) was more effective than *E. coli *and *K. pneumonia*.

**Table 1:** Minimum inhibitory concentration (MIC μgmL^-1^) of ciprofloxacin, norfloxacin, gatifloxacin and compounds 5-7 (a-c) against some common bacteria by agar dilution method

**Microorganisms**								
**Compound **	***S.aureus***	***E.coli***	***P.aeruginosa***	***K.pneumoniae***	***B.subtilis***	***S.epidermidis***	**MRSA3**
Ciprofloxacin (1)	0.195	0.012	0.0391	0.003	0.195	0.195	0.391
5a	0.391	0.049	0.781	0.012	0.391	0.391	1.563
5b	3.125	1.563	12.5	0.195	3.125	6.25	6.25
5c	0.391	0.049	3.125	0.006	0.391	0.391	6.25
Norfloxacin (2)	0.391	0.049	0.781	0.024	0.391	0.781	0.781
6a	6.25	0.195	12.5	0.049	1.563	3.125	>100
6b	1.563	0.195	6.25	0.024	1.563	1.563	3.125
6c	0.781	0.049	3.125	0.024	1.563	1.563	25
Gatifloxacin (3)	0.049	0.024	0.781	0.006	0.049	0.049	0.098
7a	0.195	1.563	100	0.098	0.195	0.098	3.125
7b	0.781	3.125	100	0.781	0.781	1.563	12.5
7c	0.098	0.781	50	0.049	0.391	0.098	3.125

In conclusion, the initiation of bulky anthracene and phenanthrene moieties on piperazine ring at C-7 position of fluoroquinolones reduced the antibacterial activities against both gram-negative and gram-positive bacteria.
